# Supplementary material for: The Immunomodulatory Effects of Fluorescein-Mediated Sonodynamic Treatment Lead to Systemic and Intratumoral Depletion of Myeloid-Derived Suppressor Cells in a Preclinical Malignant Glioma Model
Source: Cancers (Basel). 2024 Feb 15;16(4):792. doi: 10.3390/cancers16040792 (PMC10886594; doi:10.3390/cancers16040792)

Supplementary materials

Here we report the set-up of our experiment. The animal was anesthetized using tribroethanol and positioned prone on a soft pad. The transducer was positioned close to the mouse's skull, at the level of the tumor injection point. A US aqueous coupling medium was used to reduce to minimum air interference.

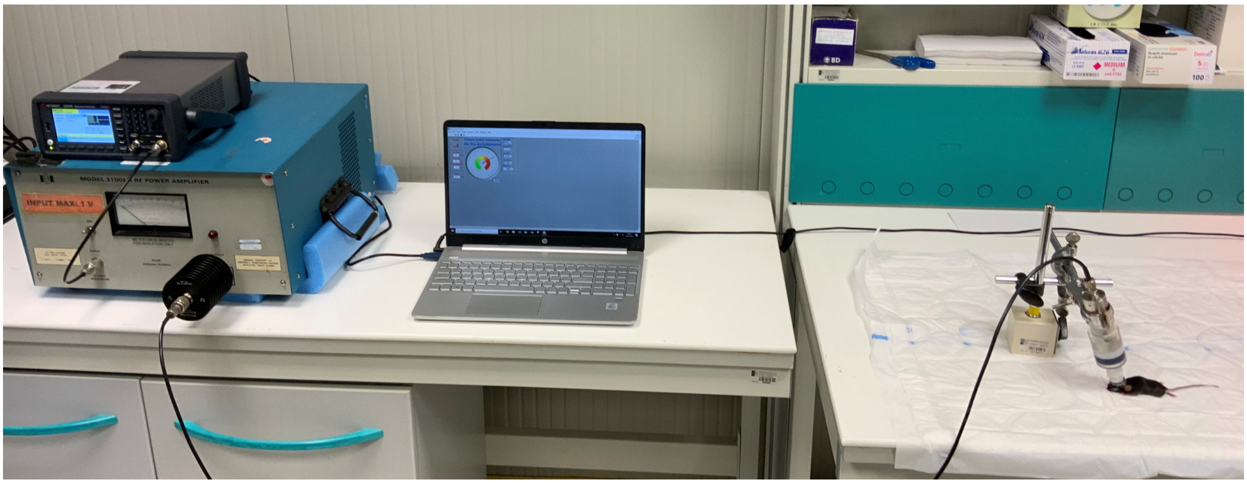

Supplement: Supplementary file 1 [file cancers-16-00792-s001.zip › cancers-2620972-supplementary.pdf]
